# Public and Private Healthcare Service Quality in Trujillo, Peru: Evidence from a SERVQUAL Survey

**DOI:** 10.3390/healthcare14060738

**Published:** 2026-03-13

**Authors:** Pedro Oloya-Salazar, Ener Alayo-Ruiz, Katia Vallejos-Salas, María Ruiton-Castillo, Johanna Peña-López, Kiara Anicama-Ramirez, Walter Rojas-Villacorta

**Affiliations:** 1 Escuela Profesional de Medicina, Facultad de Ciencias de la Salud, Universidad César Vallejo, Trujillo 13001, Peru; poloyasa@ucvvirtual.edu.pe (P.O.-S.); eralayo@ucvvirtual.edu.pe (E.A.-R.); kvallejossa1@ucvvirtual.edu.pe (K.V.-S.); mruiton@ucvvirtual.edu.pe (M.R.-C.); jpenalo@ucvvirtual.edu.pe (J.P.-L.); kianicamara@ucvvirtual.edu.pe (K.A.-R.); 2 Dirección de Investigación, Universidad César Vallejo, Trujillo 13001, Peru

**Keywords:** quality of healthcare, patient satisfaction, patient experience, healthcare evaluation, hospitals, clinics, Peru, cluster analysis

## Abstract

**Highlights:**

**What are the main findings?**

- Significant differences in perceived service quality and patient satisfaction were found between public hospitals and private clinics in Trujillo, Peru.
- Empathy and tangibles were key predictors of satisfaction, and cluster analysis identified two distinct user profiles based on service quality dimensions.

**What are the implications of the main findings?**

- The findings highlight disparities in healthcare service delivery that affect patient experience and satisfaction in public and private institutions.
- The results provide evidence to support policies and strategies aimed at strengthening equitable, patient-centered healthcare in line with SDG 3.

**Abstract:**

**Background:** Service quality and patient satisfaction are key indicators of healthcare performance, yet disparities remain between public hospitals and private clinics in Peru. Understanding these differences is essential for improving patient-centered care and advancing Sustainable Development Goal 3 (Good Health and Well-Being). The study examined how perceived service quality relates to user satisfaction in Trujillo’s private and public institutions. **Methods:** A cross-sectional study was conducted involving 480 users from public and private healthcare institutions. Service quality was assessed using the SERVQUAL model, and user satisfaction was measured with a validated Likert-scale instrument. Data did not follow a normal distribution (Kolmogorov–Smirnov test); thus, nonparametric statistics were applied. A two-step cluster analysis was additionally performed to identify user profiles based on the five quality dimensions of quality. **Results:** Participants from both health centers exhibited a range of sociodemographic profiles with regard to age, gender and income. Private clinics reported high levels of perceived service quality (95.8%) and user satisfaction (89.3%), whereas the public hospital showed moderate ratings in both dimensions. In the public setting, empathy and tangible elements emerged as significant predictors of satisfaction, while in private clinics, these same dimensions exhibited negative associations. The cluster analysis identified two distinct user profiles, with tangibles and reliability being the most influential predictors. **Conclusions:** Significant differences were observed between private and public institutions. Although service quality was positively associated with satisfaction, its explanatory power was limited, suggesting the influence of additional unmeasured factors. This study opens avenues for future research on how differentiated strategies can be scaled and adapted to strengthen public healthcare delivery in Peru, ensuring alignment with equitable and patient-centered care principles promoted by SDG 3.

## 1. Introduction

Service quality and user satisfaction are fundamental pillars of private healthcare, directly influencing perceived value, patient loyalty, and ultimately, institutional sustainability. The World Health Organization (WHO) defines quality of care as the extent to which health services increase the likelihood of desired health outcomes [1]. Within private clinics, however, a potential concern arises: the prioritization of financial interests over patient needs and satisfaction. This issue gained public attention following an accident at a shopping center in Trujillo, where several clinics conditioned emergency care for victims on upfront payment or financial guarantees. As a result, many patients were transferred to other hospitals in the region [2]. This incident suggests that, in our country, similar practices may be occurring in some private clinics, where the pursuit of profitability could influence decisions related to consultation duration, the indication of tests or procedures, cost transparency, and the willingness to provide emergency care without prior financial guarantees [3].

Two central concepts underpin healthcare evaluation: service quality and patient satisfaction. Quality refers to the attributes and processes that ensure effective, safe, and patient-centered care [4]. Satisfaction, in turn, is a multidimensional and subjective construct arising from the comparison between prior expectations and the actual healthcare experience [5].

Beyond these traditional conceptualizations, contemporary health systems increasingly frame quality and satisfaction within the value-based healthcare (VBHC) paradigm, which prioritizes achieving better health outcomes relative to costs. Within this framework, perceived quality plays a critical role in promoting treatment adherence, continuity of care, and improved clinical outcomes. In the Peruvian healthcare system, ongoing efforts to enhance efficiency, quality, and user satisfaction are consistent with this value-oriented approach. Therefore, systematically evaluating service quality dimensions provides relevant evidence aligned with current health policy priorities [6].

The current management of private healthcare systems has adopted user satisfaction as a central element in service provision, increasingly referring to individuals as “users” or “clients” rather than “patients.” This shift in terminology reflects an effort to optimize both performance and efficiency, acknowledging the business-oriented nature of private clinics. Within this framework, the National Superintendence of Health Insurance (SUSALUD, by its Spanish acronym), the regulatory body overseeing health insurance in Peru, is responsible for ensuring compliance with quality standards and for considering user satisfaction in its oversight [7]. Given the inherent difficulty in objectively measuring healthcare quality, experts often rely on indirect assessments, including economic indicators, health outcomes (such as reductions in morbidity and mortality), and ultimately, the level of user satisfaction. In recent decades, research on user satisfaction has focused primarily on how individuals perceive the care they receive, prioritizing their experience as a key factor in evaluating healthcare services [3].

Consequently, excellence in the care provided by private clinics is a key factor in securing user approval. This can be classified as service quality, which, according to the WHO, is defined as the effective application of professional skills, the optimal use of available resources, the minimization of risks to the user, and the assurance of their well-being. The user’s perception of quality has a direct impact on their overall experience and level of satisfaction with the clinic’s services [1]. Accordingly, user satisfaction is a critical factor in evaluating the quality of services provided by private clinics. This involves not only effectively addressing users’ health concerns but also considering their experience throughout the entire care process. In this regard, key elements contributing to user satisfaction have been identified, including staff friendliness, responsiveness to user needs, transparency and clarity of the information provided, and the empathy demonstrated by healthcare professionals [8].

Several international studies confirm the close relationship between service quality and patient satisfaction in healthcare settings. Coman (2021) highlighted high overall satisfaction, identifying physician behavior as the main determinant, followed by the medical team and interventions provided [9]. Similarly, Prieto (2022) reported high satisfaction levels in a dental clinic in Guayaquil, noting the influence of expectations and staff communication [10]. Friedel (2023) found a 75% satisfaction rate at Essen Hospital, emphasizing personnel quality while pointing out long waiting times and bureaucratic barriers as weaknesses [11]. Recent evidence further supports the multidimensional nature of satisfaction: Mularczyk-Tomczewska et al. (2025) identified factors such as quality of care, accessibility, financial aspects, digitization, and empathetic communication in Poland [12]; Wayesa et al. (2025) reported moderate satisfaction in Ethiopia, associated with communication skills and sociodemographic variables [13]; and Eissa et al. (2025) in Syria revealed high patient satisfaction despite neutral professional attitudes toward TQM, suggesting systemic challenges [14]. Overall, patient satisfaction emerges as a multidimensional indicator reflecting not only technical care quality but also organizational and contextual factors.

In Peru, several studies have examined user satisfaction in healthcare. Hernández-Vásquez et al. [15], using ENAHO 2018 data, found that 74.3% of MINSA users rated care as “good or very good,” though satisfaction was lower among those with chronic conditions or native language backgrounds. Barrios-Ipenza et al. [16] validated the HEALTHQUAL scale in Lima, showing significant associations in PPP hospitals between satisfaction, efficiency, and loyalty. In private clinics, Gutiérrez (2022) [17] reported 78% high service quality and 55% “excellent” experiences, while Estrada and Zumaeta (2022) [18] in Piura observed strong correlations (rho = 0.83) between quality and satisfaction, despite empathy and reliability gaps. Febres (2020) [19] in Huancayo found 60% satisfaction despite infrastructure deficiencies, highlighting safety and empathy as strengths. Martin Navarro (2021) [20] emphasized punctuality and reduced waiting times, and Sánchez (2022) [21] in Trujillo reported 60.3% satisfaction, with high scores in safety and empathy but dissatisfaction in tangibles and responsiveness.

A comparison of private and public healthcare institutions reflects the structural reality of the Peruvian health system, where users choose providers based on perceived quality, accessibility, and trust. Private institutions are often associated with personalized care and modern infrastructure, whereas public hospitals serve more socioeconomically diverse populations with limited resources. Examining these differences is essential to identify gaps in satisfaction and expectations, particularly in intermediate cities like Trujillo, where comparative evidence remains limited.

In Peru, public and private healthcare differ across multiple dimensions. Public hospitals, larger in size and coverage, often face constraints in human and financial resources, whereas smaller private clinics benefit from greater capital investment and modern infrastructure. Public institutions operate under hierarchical state structures, while private providers are managed autonomously with market-oriented leadership and innovation. Moreover, care processes in public hospitals are standardized and tend to be less limited in information systems, in contrast to private clinics, which emphasize technological integration and quality protocols. These structural and functional differences shape users’ perceptions of quality and satisfaction, thereby justifying studies such as the need for comparative studies of the present one [22,23,24].

In a context where healthcare provision has become increasingly commodified, understanding users’ perceptions of service quality is essential to assess the impact of clinical strategies on patient experience. Various aspects of care, shaped by the economic priorities of private institutions, can significantly influence user satisfaction. Doubova et al. (2024) [25] demonstrate that perceived quality is a decisive factor in choosing private services, even when this entails higher out-of-pocket expenses, underscoring the need to rethink care models from a patient-centered perspective. Evidence on perceived strengths and weaknesses allows for concrete recommendations that can guide private clinics toward more transparent, ethical, and population-responsive systems.

This study is the first in Peru to use cluster analyses to examine both perceived service quality and user satisfaction in public and private healthcare institutions simultaneously. In this context, the following research question arises: To what extent do the dimensions of service quality influence user satisfaction in public and private healthcare institutions in Trujillo? This novel approach enables a differentiated user profile to be identified and contributes to evidence-based service design in mid-sized Peruvian cities. Accordingly, this study aims to determine the relationship between service quality and user satisfaction in a private clinic and a public hospital in the city of Trujillo throughout July 2025. This objective aligns with SDG 3, which emphasizes effectiveness, safety, patient-centeredness, timeliness, equity, integration, and efficiency as defined by the WHO [26]. The hypothesis posits that higher perceived service quality is associated with higher user satisfaction and that this association is stronger for public institutions than private ones due to structural and organizational disparities.

## 2. Materials and Methods

### 2.1. Type, Approach and Research Design

This study was framed within a basic research approach with a quantitative focus, using a non-experimental cross-sectional design with a descriptive–correlational scope. This approach allowed for the analysis of relationships between variables without manipulating their behavior, making it suitable for exploring perceptions in natural contexts. By avoiding direct intervention, the study preserves internal validity within the limits of the design and provides a contextualized representation of the phenomenon under investigation, consistent with the current state of knowledge.

### 2.2. Variable

In this study, the dependent variable was user satisfaction, understood as the degree of perceived conformity by patients regarding the services received [3]. The independent variable was service quality, operationalized through its constituent dimensions, namely reliability, responsiveness, empathy, assurance, and tangibles, based on the SERVQUAL model [27].

### 2.3. Population, Sample, and Sampling

The study population consisted of users who attended two private clinics and one public specialized hospital in the city of Trujillo. Participants included individuals over 18 years of age who had utilized healthcare services at these institutions. Minors and individuals who had not required medical attention in the past year were excluded, as their opinions would not align with the study’s objectives. Likewise, visitors and tourists were excluded, given that their experiences would not reflect the perspective of the resident population in Trujillo.

A non-probabilistic convenience sampling method, a widely used approach in clinical research, was employed. It was selected based on participant accessibility and the operational feasibility of the study [28]. This strategy enabled efficient data collection in terms of time and resources, considering the logistical and financial constraints inherent to the research [29]. Although this type of sampling does not guarantee statistical representativeness of the entire population, it is appropriate for descriptive studies aiming to identify preliminary patterns in specific contexts [30].

The sample size was calculated at 480 participants, using a 95% confidence level (Z = 1.96), a 5% margin of error (e), and an estimated population proportion of 50% (p). The required sample proportion was determined using the following formula:

n = Z^2^ × p × (1 − p)/e^2^ (1)

### 2.4. Data Collection Techniques

Data collection was conducted using a paper-based survey, a widely employed technique in cross-sectional studies. The surveys were administered in situ, and each interview took around 8–10 min. Users were approached immediately after exiting the consultation areas of both institutions. This strategy minimized recall bias and ensured that responses reflected the immediate care experience (perceptions, attitudes, and satisfaction) [31]. The instrument used was a structured questionnaire, administered during July 2025. This process was carried out following the formal submission of the research protocol to the Institutional Ethics Committee, in accordance with ethical principles of voluntariness, informed consent, and confidentiality. Although the ethical approval was issued subsequently, the study adhered to the required standards (Approval N°. 004P-CEI-EPM-UCV-2025).

This instrument was adapted from the study of Vigo (2020) [32] to assess service quality and user satisfaction based on the SERVQUAL model. This model provides a standardized framework for measuring the evolution of perceived quality in healthcare services [33]. The questionnaire was structured using a Likert scale (1 = never, 2 = almost never, 3 = sometimes, 4 = almost always, 5 = always). The first questionnaire, focused on service quality, consisted of 25 items covering five core dimensions: tangibles, reliability, responsiveness, assurance, and empathy. The second questionnaire, aimed at measuring user satisfaction, included 14 items and assessed three dimensions: perceived performance, expectations, and satisfaction. The questions addressed aspects such as users’ perceptions of price accessibility, reliability of laboratory results, and staff friendliness. Total scores for service quality (25–125) and user satisfaction (14–70) were categorized as low, medium or high using empirical cut-off points derived from the observed distribution in the sample, applying tertiles as reference thresholds. This classification enabled comparative analysis between institutions. Moreover, both questionnaires yielded Cronbach’s alpha values of 0.893 and 0.870, respectively, indicating “excellent” internal consistency [32]. These results underscore the precision and reliability of the measurements used in the study.

### 2.5. Data Analysis

Descriptive and inferential statistics were used for data analysis. IBM SPSS Statistics version 27 was employed as the primary software, with data initially organized in Microsoft Excel 2024. For analytical purposes, age was grouped into three categories: under 40 years, 40–59 years, and 60 years and older. Participants who were exactly 40 years old were included in the 40–59 age group. Monthly income was classified as less than 1000 PEN, 1000–2500 PEN, 2500–3500 PEN, or greater than 3500 PEN. Participants with an income of exactly 2500 PEN were placed in the 2500–3500 group to maintain consistent grouping.

The normality of the data was verified exclusively using the Kolmogorov–Smirnov test, which confirmed that the distribution did not meet the assumption of normality. Consequently, nonparametric tests were applied in the analyses. Comparisons between healthcare institutions were conducted using the Mann–Whitney U test. The association between service quality dimensions and user satisfaction was evaluated using Spearman’s rho. Multiple linear regression models were fitted separately for each institution to identify predictors of satisfaction. Additionally, two-step cluster analysis was applied to segment user profiles based on the five SERVQUAL dimensions (D1–D5). Cluster quality was evaluated using the silhouette coefficient. Values ≥ 0.50 indicate acceptable structure, and the value obtained in this study (S = 0.70) reflects good cohesion and separation between clusters [33].

A post hoc power analysis conducted with G*Power version 3.1.9.7 (α = 0.05, two-tailed) confirmed that the correlation analyses had adequate statistical power. The total sample size of 480 achieved a power of 0.99 for detecting small-to-medium effect sizes. Subgroup analyses also demonstrated high power, with values of 0.99 for the public institution (ρ = 0.27) and 0.91 for the private institution (ρ = 0.21).

### 2.6. Ethical Considerations

This research was conducted in strict accordance with the ethical principles outlined in the Declaration of Helsinki and the Peruvian General Health Law (Law No. 26842) [34,35]. The study protocol was submitted for ethical review and approved by the Research Ethics Committee of the School of Medicine at Universidad César Vallejo (Approval N°. 004P-CEI-EPM-UCV-2025). Although formal approval by the ethics committee was issued retrospectively due to administrative reasons, the protocol had been submitted beforehand, and all data collection activities were conducted in accordance with the ethical principles of the Declaration of Helsinki and the Peruvian General Health Law. Participation was entirely voluntary. All participants received clear information about the study’s objectives, procedures, expected duration, potential risks, and data confidentiality measures. Informed consent was obtained from each participant before data collection began. For digital questionnaires, participants provided consent by actively selecting the approval checkbox at the beginning of the survey. This constituted documented electronic consent. No personal identifiers were collected, and all data were anonymized to ensure participant privacy. The study adhered to the principles of respect for persons, beneficence, and justice in all procedures.

## 3. Results

### 3.1. Sociodemographic Distribution of Healthcare Users

Table 1 shows that among the sample of 480 participants, both the public hospital and the private clinics predominantly serve female users, with 55.8% and 69.0%, respectively. Most individuals fall within the 40–59 age range, accounting for 52.1% in hospitals and 44.2% in clinics. Regarding income levels, a notable difference is observed: users of the public hospital predominantly earn between 1000 and 2500 PEN (Peruvian nuevo sol/Peruvian sol: current currency), while the two private clinics attract a significant proportion of individuals with monthly incomes exceeding 3500 PEN.

### 3.2. Institutional Comparison of Service Quality and Satisfaction

Table 2 shows the distribution of perceived service quality and user satisfaction according to the type of healthcare institution. In the public hospital, most users rated service quality as medium, followed by low, with no reports of high quality. In contrast, the majority of users in private clinics rated service quality as high, with a small percentage reporting medium quality and none reporting low quality. Regarding user satisfaction, the public hospital showed a predominance of medium-level satisfaction, followed by low, and only one case of high satisfaction. In private clinics, most users reported high satisfaction, followed by medium, with no reports of low satisfaction. The nonparametric Mann–Whitney U test revealed statistically significant differences in both perceived service quality (U = 10,500; Z = −18.955; *p* < 0.001) and user satisfaction (U = 250.500; Z = −18.591; *p* < 0.001) between the public hospital and the private clinics.

### 3.3. Correlation Between Service Quality Dimensions and User Satisfaction

Table 3 presents the correlations between perceived service quality (overall and by dimension) and user satisfaction in a public hospital in the city of Trujillo. Positive and statistically significant associations were identified in all cases. Overall service quality showed a low correlation with satisfaction (rho = 0.27; 95% CI: 0.14–0.38; *p* < 0.001), corresponding to a coefficient of determination (R^2^) of 0.078. This indicates that perceived service quality explains approximately 7.8% of the variability in user satisfaction. Among the dimensions, the strongest correlations were observed with assurance (D4: rho = 0.30; 95% CI: 0.17–0.41; *p* < 0.001) and empathy (D5: rho = 0.27; 95% CI: 0.15–0.39; *p* < 0.001), while associations with reliability, tangibles, and responsiveness were significant but of lower magnitude.

Table 4 shows that in the two private clinics, perceived service quality also demonstrated a positive, albeit low, correlation with user satisfaction (rho = 0.21; 95% CI: 0.07–0.33; *p* = 0.001), equivalent to a coefficient of determination (R^2^) of 0.039. This suggests that perceived service quality explained approximately 3.9% of the variability in user satisfaction. Significant correlations were found with reliability (D2: rho = 0.13; 95% CI: 0.00–0.24; *p* = 0.043), responsiveness (D3: rho = 0.18; 95% CI: 0.05–0.30; *p* = 0.006), and assurance (D4: rho = 0.20; 95% CI: 0.07–0.32; *p* = 0.002). In contrast, correlations with tangibles (D1: rho = 0.01; *p* = 0.860) and empathy (D5: rho = 0.05; *p* = 0.412) were not statistically significant.

### 3.4. Predictive Models of Satisfaction Using Multiple Linear Regression

Table 5 presents the multiple linear regression results for the public hospital. The model was statistically significant (*p* < 0.001), with a modest fit (R^2^ = 0.100; adjusted R^2^ = 0.080). Empathy (D5: B = 0.392; *p* = 0.002) and tangibles (D1: B = 0.308; *p* = 0.046) emerged as positive and significant predictors of user satisfaction. In contrast, reliability, responsiveness, and assurance did not show statistically significant effects (*p* > 0.05).

Table 6 displays the multiple linear regression results for the two private clinics. The model was statistically significant (*p* = 0.007), though with lower explanatory power (R^2^ = 0.072; adjusted R^2^ = 0.048). In this case, tangibles (D1: B = −0.659; *p* = 0.045) and empathy (D5: B = −0.709; *p* = 0.030) were negatively and significantly associated with user satisfaction. The dimensions of reliability (D2), responsiveness (D3), and assurance (D4) did not reach statistical significance (*p* > 0.05).

### 3.5. User Segmentation Through Two-Step Cluster Analysis

**Figure 1:**
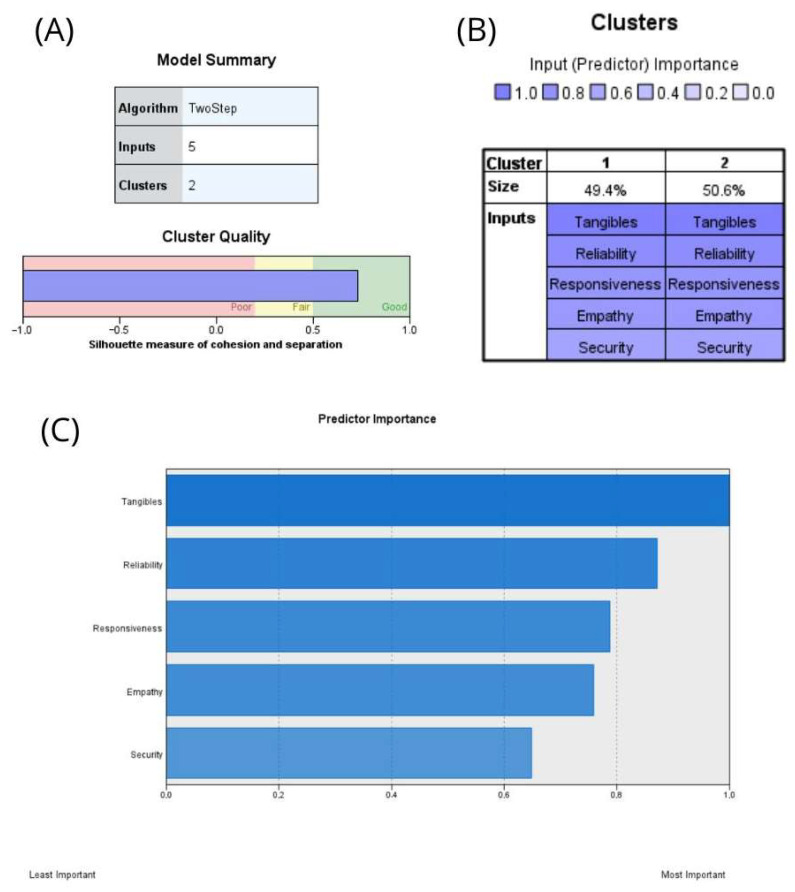
Cluster analysis results. (**A**) Model quality (silhouette = 0.7). (**B**) Cluster distribution and predictor importance. (**C**) Predictor importance.

The results of the cluster analysis are presented in three stages: first, model quality was evaluated (Figure 1A); second, group distribution was examined (Figure 1B); and finally, the predictors and characteristics of each cluster were analyzed (Figure 1C and centroids). The two-step clustering algorithm identified two distinct user groups based on the five dimensions of service quality. The average silhouette coefficient was 0.7, indicating a good and robust segmentation, which confirms the validity and coherence of the clusters. As shown in Figure 1B, the two groups were relatively balanced in size, comprising 49.4% and 50.6% of users, respectively. Predictor importance results (Figure 1C) revealed that tangibles (D1: 1.00) and reliability (D2: 0.87) were the most influential dimensions in differentiating the clusters, followed by responsiveness (D3: 0.79), empathy (D5: 0.76), and assurance (D4: 0.65). Centroid values (Table A1) showed marked differences in satisfaction levels: Cluster 1 exhibited mean scores close to 13 across all dimensions, corresponding to a profile of users with low satisfaction, whereas Cluster 2 recorded average scores between 20 and 22, representing a profile of highly satisfied users. This 7–8 point difference across dimensions reinforces the clear separation between groups and the strength of the clustering model.

The identified clusters reflect differentiated profiles in the perception of service quality across the five evaluated dimensions (tangibility, reliability, responsiveness, assurance, and empathy). This grouping allows for distinguishing users with consistently high ratings across all dimensions from those with lower evaluations, particularly in aspects related to responsiveness and empathy, which may guide targeted interventions to strengthen these critical areas.

## 4. Discussion

The sociodemographic profile (Table 1) of users in both private clinics and the public hospital reveals a predominance of female adults aged 40 to 59 years. This result is consistent with findings of Febres-Ramos and Mercado-Rey (2020) [19], who reported a higher female participation rate (61%) and elevated demand among this age group in Huancayo. This suggests that middle-aged women continue to be the primary users of outpatient and hospital services, likely reflecting their central role in family health management. Income-level differences are also evident in public hospitals; low to middle incomes prevail (37.9% earning between 1000 and 2500 PEN), whereas private clinics show a notable concentration of users with monthly incomes exceeding 3500 PEN (29.6%). This trend is consistent with Araujo Verde (2022) [36], who emphasizes that access to private clinics is closely linked to purchasing power, given the greater comfort, shorter waiting times, and personalized services they offer. These differences reflect not only the influence of economic status on the choice of healthcare facility but also on users’ expectations and perceptions of care. Importantly, they reinforce the objective of this study: to analyze how service quality dimensions influence user satisfaction in public and private healthcare institutions in Trujillo.

The findings revealed statistically significant differences in perceived service quality (U = 10,500; Z = −18.955; *p* < 0.001) and user satisfaction (U = 250.500; Z = −18.591; *p* < 0.001) between the public hospital and private clinics (Table 2). In practical terms, the public hospital was predominantly rated as “medium” in both quality and satisfaction, whereas private clinics were mostly rated as “high.” This contrast suggests that factors such as better infrastructure, shorter waiting times, and personalized care in private clinics contribute to a more favorable user experience. These results are consistent with studies conducted in Indonesia [37] and Turkey [38], where perceived institutions typically demonstrate better performance in user-perceived quality. Similarly, local research has documented that in some public hospitals in Peru, more than 50% of users rate the care as “regular,” attributing this to limitations in human resources and administrative management [39,40,41].

However, not all contexts follow the same pattern. Umeokonkwo et al. (2018) [42] observed in Nigeria that public facilities may, in some contexts, achieve higher satisfaction than private ones, depending on user expectations and organizational culture. Additionally, studies in Peruvian public hospitals emphasize that deficiencies in human interaction and administrative management, particularly in emergency services, are key drivers of dissatisfaction, reinforcing the need to strengthen these dimensions [43]. In Ecuador, expectations of service quality have been shown to center on assurance, punctuality, and personalization [44]. In Colombia, empathy and safety were the most valued aspects during the COVID-19 pandemic, while responsiveness was the most affected aspect [45]. The present study supports this interpretation, suggesting that the institutional context plays a decisive role in the perception of quality and not just the classification between public and private.

In the public hospital (Table 3), perceived service quality demonstrated a low correlation with satisfaction (rho = 0.27; R^2^ = 7.8%). The strongest associations were found for the dimensions of assurance (D4) and empathy (D5), indicating that users highly value feeling safe and receiving supportive, respectful attention. Gonzaga (2024) similarly reported that reliability and assurance were directly linked to higher satisfaction levels in public hospitals within Peru [46]. These findings reinforce the importance of relational and emotional components in environments where resource limitations may heighten user vulnerability.

In private clinics (Table 4), correlations were also positive but weaker (rho = 0.21; R^2^ = 3.9%). Reliability, responsiveness, and assurance showed significant associations, whereas tangible elements and empathy did not. These findings align with those of Aguilar-Ramos et al. (2022), who identified reliability as the most influential factor in patient satisfaction among EsSalud users [47]. In contrast, dimensions such as tangible elements and empathy were not significant in the private context, differing from the findings of Al-Neyadi et al. (2018) in hospitals in the United Arab Emirates, where responsiveness was considered the least relevant factor [48]. Taken together, the results show that while perceived quality is associated with satisfaction in both public hospitals and private clinics, the specific dimensions driving this relationship vary by institution type, reflecting the influence of organizational context and user expectations. Importantly, the explanatory power of these associations remains limited, reinforcing that patient satisfaction is shaped by multiple unmeasured factors beyond SERVQUAL dimensions. These findings should therefore be interpreted as exploratory evidence of perceptual differences rather than causal or structural superiority of one sector over the other.

The relatively low explanatory power observed in these associations (R^2^ < 10%) may reflect the inherently multidimensional nature of patient satisfaction in healthcare. Beyond perceived service quality, satisfaction is also influenced by factors such as waiting times, clinical outcomes, communication processes, and previous healthcare experiences. Therefore, SERVQUAL dimensions capture only part of the broader process through which patients evaluate healthcare services.

The regression results for the public hospital (Table 5) showed that tangibles and empathy were significant positive predictors of satisfaction, explaining 10% of its variability (R^2^ = 0.100). Although modest, this explanatory power should be interpreted as evidence of the multifactorial nature of patient satisfaction rather than model weakness. Satisfaction is shaped not only by perceived service quality but also by unmeasured determinants such as waiting times, clinical outcomes, continuity of care, and organizational processes. Within this context, the SERVQUAL dimensions provide partial yet meaningful insights into user perceptions in the public institution. These findings are consistent with research conducted at Al-Baha General Hospital in Saudi Arabia, where similar dimensions had the greatest impact on patient satisfaction [49]. Importantly, the limited variance explained reinforces the need for cautious interpretation and highlights the importance of considering broader contextual and systemic factors in future studies.

These findings also illustrate the contextual instability frequently reported for the SERVQUAL model. The relative importance of its dimensions may vary depending on institutional context and patient expectations. In private healthcare settings, attributes such as infrastructure and empathetic treatment may be perceived as baseline standards, which can reduce their direct influence on satisfaction levels.

In private clinics (Table 6), although the model was statistically significant, empathy and tangible elements emerged as significant negative predictors in the regression model. This finding can be interpreted as a baseline expectation effect: in private healthcare settings, infrastructure quality and empathetic treatment are regarded as established standards; therefore, their presence does not necessarily enhance satisfaction, whereas their absence may negatively affect it. Accordingly, these factors may function as necessary but not sufficient conditions for satisfaction, while other unmeasured variables may more adequately explain satisfaction in this context. This interpretation contrasts with findings from private hospitals in Tehran, where multiple dimensions explained nearly 45% of satisfaction variability and the physical environment was not significant [50]. In Peru, previous studies have likewise shown that satisfaction is associated with sociodemographic characteristics and service-related factors [51,52,53]. In private clinics, satisfaction relies on more complex and elevated expectations. The results indicate that SERVQUAL dimensions may not behave consistently across contexts; the negative coefficients for tangibles and empathy may reflect ceiling effects, differentiated expectations, or measurement artifacts and should therefore be interpreted with caution.

Cluster analysis (Figure 1) identified two clearly differentiated user profiles: one with low satisfaction levels and another with consistently high scores across all SERVQUAL dimensions. The model’s robustness was supported by a silhouette coefficient of 0.7, indicating strong cohesion within clusters and clear separation between them. Notably, this represents the first application of cluster analysis to segment healthcare users based on service quality perceptions and satisfaction within the Peruvian context, providing a methodological contribution that extends beyond traditional SERVQUAL evaluations.

The centroids also revealed a clear contrast between dissatisfied and highly satisfied users, reinforcing the validity of the segmentation and its relevance for understanding heterogeneity in patient experience. Tangible elements and reliability were the most influential predictors in the formation of these groups, highlighting the importance of infrastructure, resource availability, and fulfillment of service promises in shaping user perceptions. Similar findings have been reported in other contexts. In Norway, a study involving over 23,000 hospitalized patients identified six clusters, including one with consistently low evaluations and another with highly negative perceptions linked to medical errors [54]. In the same country, a study in palliative care reported three patient profiles with varying levels of perceived quality and satisfaction, associated with both individual and organizational factors [55]. Complementarily, in Finland, hospitalized patients were classified into four satisfaction profiles, ranging from dissatisfied to highly satisfied, with variations based on age, health status, and admission conditions [56]. Collectively, these studies support the validity of the segmentation obtained in our research and underscore the role of tangible elements and reliability as key factors in differentiating user experience in healthcare services.

Although the study was conducted in a single city using convenience sampling, which limits statistical generalizability, the findings may still provide useful insights for similar urban contexts in Peru and other Latin American countries where public and private healthcare systems share comparable structural and organizational characteristics. In this context, the results also reinforce the need to understand healthcare quality as a multidimensional construct that extends beyond perceived service quality. While perception-based tools such as SERVQUAL offer valuable insights into patient experience, they represent only one component of overall healthcare quality, which also encompasses dimensions such as clinical effectiveness, patient safety, and accessibility, highlighting the importance of adopting more comprehensive approaches when evaluating healthcare performance.

### 4.1. Practical Implications

The findings are highly relevant and have direct and significant implications for effective management and contemporary health policies. A comprehensive understanding of the needs and expectations of healthcare service users is imperative for achieving substantial improvements. It is noteworthy that public hospitals, in particular, stand to benefit from increased investment in key infrastructure and the implementation of relational care initiatives. These measures are expected to yield substantial improvements in patient experience, as evidenced by a reduction in wait times. Conversely, training programs that emphasize empathy, effective communication, and patient-centered care are likely to further improve the aspects that influence overall user satisfaction in a straightforward manner. In private clinics, where users have elevated expectations, intervention programs should prioritize continuity of care and differentiation from other services.

### 4.2. Limitations and Future Research

This study has several limitations that constrain the generalizability of its findings. Its cross-sectional design prevents establishing causal relationships, and the use of non-probabilistic convenience sampling may introduce self-selection bias. Data were collected in one public hospital and two private clinics in Trujillo, which restricts the scope of inference and may not reflect perceptions in other regions of Peru. Therefore, the findings should be interpreted as institution-specific rather than representative of the entire public or private healthcare sector in Peru. In private clinics, ceiling effects were evident, with most ratings clustered at the highest levels, limiting variability and raising questions about whether the SERVQUAL instrument is sufficiently sensitive in contexts with sharply different expectations and socioeconomic profiles. Moreover, satisfaction may conceptually overlap with certain SERVQUAL dimensions, which could partially inflate the observed associations. Despite these limitations, the study contributes methodologically by applying cluster analysis for the first time in a Peruvian context and highlights the need for future longitudinal designs, probabilistic sampling, and more robust models.

## 5. Conclusions

The current research provides preliminary evidence of perceptual differences in user perceptions of service quality and satisfaction between public and private healthcare facilities in Trujillo. These differences reflect the unique sociodemographic characteristics and expectations of the communities they serve. In the public sector, user satisfaction appears to be closely tied to the condition of the physical surroundings and the quality of personal interactions. This highlights the need to improve infrastructure and patient-focused communication methods. Although a positive correlation was found between perceived quality and satisfaction, the model’s limited explanatory power (R^2^ < 10%) suggests that other contextual and organizational factors significantly contribute but were not included in this analysis. The cluster results further demonstrate the diversity of user experiences and highlight opportunities for more customized improvement strategies. However, given that this was a cross-sectional study using non-probabilistic sampling, causal relationships cannot be established, and the results are not generalizable. Future studies would benefit from longitudinal designs, probabilistic sampling, and more comprehensive or mixed-methods approaches to better investigate the factors influencing user satisfaction and support public-sector quality improvement initiatives in line with SDG 3.

Consequently, the findings should be interpreted as institution-specific to the facilities studied in Trujillo and not as representative of the entire public or private healthcare sector in Peru.

## Figures and Tables

**Table 1:** Characteristics of users attending private and public institutions in Trujillo (Peru).

Characteristics		Public Hospital	Private Clinic
		*n* (%)	*n* (%)
Age	<40 years	72 (30.0)	98 (40.5)
	40–59 years	125 (52.1)	106 (44.2)
	≥60 years	43 (17.9)	36 (15.0)
Gender	Male	116 (44.2)	96 (40.0)
	Female	124 (55.8)	144 (69.0)
Salary income	<1000	35 (14.6)	25 (10.4)
	1000–2500	91 (37.9)	80 (33.3)
	2500–3500	86 (35.8)	64 (26.7)
	>3500 PEN *	28 (11.7)	71 (29.6)
Total		240 (100.0)	240 (100.0)

Notes: * PEN: Peruvian currency. At the time of the study, 1 USD = 3.57 PEN. For reference, income categories correspond approximately to: <1000 PEN (<280 USD); 1000–2500 PEN (280–700 USD); 2500–3500 PEN (700–980 USD); >3500 PEN (>980 USD).

**Table 2:** Distribution of perceived quality of service and user satisfaction by type of institution.

Variable	Level	Public Hospital *n* (%)	Private Clinic *n* (%)	Total *n* (%)	*p*-Value *
Service Quality	High	0 (0.0)	231 (95.8)	231 (47.9)	<0.001
	Medium	211 (87.9)	9 (3.8)	220 (45.8)	
	Low	29 (12.1)	0 (0.0)	29 (6.0)	
	Total	240 (100.0)	240 (100.0)	480 (100.0)	
Satisfaction	High	1 (0.4)	214 (89.3)	215 (44.8)	<0.001
	Medium	233 (87.9)	26 (10.8)	259 (54.0)	
	Low	6 (2.5)	0 (0.0)	6 (1.3)	
	Total	240 (100.0)	240 (100.0)	480 (100.0)	

Notes: * Mann–Whitney U test: Service quality (U = 10,500; Z = −18.955); User satisfaction (U = 250,500; Z = −18.591). All differences were statistically significant (*p* < 0.001).

**Table 3:** Relationship between perceived service quality and user satisfaction in the public hospital (*n* = 240).

Variable	Spearman Correlations	95% CI	*p*-Value
Service Quality	0.27	0.14–0.38	<0.001
D1: Tangible	0.21	0.09–0.34	0.001
D2: Reliability	0.18	0.05–0.30	0.007
D3: Responsiveness	0.15	0.02–0.28	0.020
D4: Assurance	0.30	0.17–0.41	<0.001
D5: Empathy	0.27	0.15–0.39	<0.001

Notes: 95% CI = 95% confidence interval. D1, D2, D3, D4, and D5: dimensions of quality of service. R^2^ = 7.8% (determination coefficient).

**Table 4:** Relationship between perceived service quality and user satisfaction in the two private clinics (*n* = 240).

Variable	Spearman Correlations	95% CI	*p*-Value
Service Quality	0.21	0.07–0.33	0.001
D1: Tangible	0.01	−0.19–0.14	0.860
D2: Reliability	0.13	0.00–0.24	0.043
D3: Responsiveness	0.18	0.05–0.30	0.006
D4: Assurance	0.20	0.07–0.32	0.002
D5: Empathy	0.05	−0.08–0.18	0.412

Notes: 95% CI = 95% confidence interval. D1, D2, D3, D4, and D5: dimensions of quality of service. R^2^ = 3.9% (determination coefficient).

**Table 5:** Service quality factors associated with user satisfaction in the public hospital.

Variable	B (95%CI)	Beta	t	*p*-Value
D1: Tangible	0.308 (0.005–0.610)	0.140	2.005	0.046
D2: Reliability	0.085 (−0.224–0.395)	0.040	0.543	0.588
D3: Responsiveness	0.141 (−0.177–0.460)	0.061	0.875	0.382
D4: Assurance	0.009 (−0.172–0.190)	0.007	0.102	0.919
D5: Empathy	0.392 (0.141–0.644)	0.212	3.073	0.002

Notes: Dependent variable: user satisfaction in a public hospital. The regression model was statistically significant (*F*(5, 234) = 5.175; *p* < 0.001) and explained 10.0% of the variance (R^2^ = 0.100; R^2^ adjusted = 0.080).

**Table 6:** Service quality factors associated with user satisfaction in the private clinics.

Variable	B (95%CI)	Beta	t	*p*-Value
D1: Tangible	−0.659 (−1.302–−0.016)	−0.261	−2.020	0.045
D2: Reliability	−0.562 (−1.204–0.079)	−0.226	−1.727	0.086
D3: Responsiveness	−0.335 (−0.941–0.271)	−0.147	−1.090	0.277
D4: Assurance	−0.287 (−0.885–0.311)	−0.134	−0.946	0.345
D5: Empathy	−0.709 (−1.350–−0.067)	−0.329	−2.177	0.030

Notes: Dependent variable: user satisfaction in private clinics. The regression model was statistically significant (*F*(6, 233) = 3.014; *p* = 0.007) and explained 7.2% of the variance (R^2^ = 0.072; adjusted R^2^ = 0.048).

## Data Availability

The datasets supporting the conclusions of this article are available within the article itself.

## Abbreviations

The following abbreviations are used in this manuscript:

SDG 3	Sustainable Development Goal 3
SERVQUAL	Service Quality
WHO	World Health Organization
MINSA	Ministerio de Salud (Acronyms in Spanish)

## Appendix A

**Table A1:** Centroid values of service quality dimensions across user clusters.

Cluster	Tangibles (Mean ± SD)	Reliability (Mean ± SD)	Responsiveness (Mean ± SD)	Security (Mean ± SD)	Empathy (Mean ± SD)
**1**	13.35 ± 1.93	13.27 ± 1.92	13.46 ± 1.83	12.69 ± 3.09	13.33 ± 2.25
**2**	21.83 ± 2.04	20.56 ± 2.00	20.33 ± 2.21	20.45 ± 2.32	20.75 ± 2.30
**Combined**	17.65 ± 4.68	16.96 ± 4.14	16.94 ± 3.99	16.62 ± 4.74	17.09 ± 4.36
